# A chatbot based question and answer system for the auxiliary diagnosis of chronic diseases based on large language model

**DOI:** 10.1038/s41598-024-67429-4

**Published:** 2024-07-25

**Authors:** Sainan Zhang, Jisung Song

**Affiliations:** Graduate School of Communication Design, Hanyang University, ERICA Campus, Ansan, 15588 Republic of Korea

**Keywords:** Artificial intelligence, Chatbots, Large models, CUQ test, Deep learning, User experience, Health services, Health care, Health occupations, Computer science, Information technology

## Abstract

In recent years, artificial intelligence has made remarkable strides, improving various aspects of our daily lives. One notable application is in intelligent chatbots that use deep learning models. These systems have shown tremendous promise in the medical sector, enhancing healthcare quality, treatment efficiency, and cost-effectiveness. However, their role in aiding disease diagnosis, particularly chronic conditions, remains underexplored. Addressing this issue, this study employs large language models from the GPT series, in conjunction with deep learning techniques, to design and develop a diagnostic system targeted at chronic diseases. Specifically, performed transfer learning and fine-tuning on the GPT-2 model, enabling it to assist in accurately diagnosing 24 common chronic diseases. To provide a user-friendly interface and seamless interactive experience, we further developed a dialog-based interface, naming it Chat Ella. This system can make precise predictions for chronic diseases based on the symptoms described by users. Experimental results indicate that our model achieved an accuracy rate of 97.50% on the validation set, and an area under the curve (AUC) value reaching 99.91%. Moreover, conducted user satisfaction tests, which revealed that 68.7% of participants approved of Chat Ella, while 45.3% of participants found the system made daily medical consultations more convenient. It can rapidly and accurately assess a patient’s condition based on the symptoms described and provide timely feedback, making it of significant value in the design of medical auxiliary products for household use.

## Introduction

Chronic diseases are increasingly receiving attention on a global scale. According to statistics, nearly 25% of adults suffer from one or more chronic conditions, making chronic diseases a major challenge facing global health. Not only do chronic diseases result in poor health, disability, and even death, but they also account for a substantial portion of healthcare expenditures^1^. Therefore, the early diagnosis of chronic diseases becomes critically important^2^. Prompt intervention is required to prevent further progression of the disease. With changes in lifestyle and the impact of the pandemic, the incidence of chronic diseases is on the rise. Consequently, the future demand for health management as well as expenditures on healthcare systems are also gradually increasing^3^. At the same time, the demand for healthcare resources such as hospitals and doctors has also significantly increased. Patients need to spend more time waiting for medical treatment, and doctors are facing immense workloads and patient volumes. However, to reduce both time and financial costs, an increasing number of people are leaning towards contactless consultations and seeking medical advice online^4^. In a home setting, when experiencing physical discomfort, people are more inclined to conduct online searches for medical advice^5^. Indeed, the complexity and uncertain reliability of online information present a potential problem. Therefore, there is an urgent need for remote medical interventions in the management of chronic diseases. Monitoring provided by remote healthcare service organizations can help in the early identification of symptoms and enable rapid responses to disease exacerbation^6^. Developing a system that can accurately assist in the diagnosis of chronic diseases becomes especially important.

Telemedicine is a popular aspect of electronic medical applications and has seen increased usage with the development of mobile healthcare technologies. This growth has established the significance and value of remote interventions in the management of health services^7,8^. These technological solutions have the potential to make patients’ lives easier. For example, within clinical decision support systems (CDSS), models such as naive Bayes, neural networks, and K-nearest neighbors (KNN) are utilized to diagnose COVID-19^9^. The VP-expert diagnostic system is specifically designed for populations in underdeveloped regions, aiming to rapidly diagnose diabetes and enhance the diagnostic efficiency of physicians^10^. The recommendation system for iron deficiency anemia, based on clinical oncology, serves as a CDSS, used for diagnosing and managing its treatment^11^. Furthermore, the smart tracking system for acute respiratory infections utilizes mobile health technologies to infer new facts from medical data collected during examinations of pediatric patients^12^. The use of CDSS to assist in enhancing the diagnostic efficiency of healthcare providers has become an established fact.

Currently, as the achievements of artificial intelligence in various domains have been effectively validated, deep learning has also exhibited exceptional performance in a variety of CDSS^13,14^. The concept of deep learning was introduced in 2006^15^. The primary characteristic of deep learning is unsupervised feature learning, which provides a novel solution for dealing with abstract concepts in human thought. Deep learning algorithms have been effectively applied in numerous domains, including image retrieval and web-based knowledge retrieval^16,17^. For example, expert diagnostic systems for the automatic identification of asthma and chronic obstructive pulmonary disease (COPD) based on deep learning can interpret respiratory sounds recorded by a stethoscope, assisting doctors with remote access. Such systems can provide more timely and accurate diagnoses, offering a quicker and more efficient approach, especially for patients in resource-poor areas or those who cannot receive immediate medical attention^18–20^. Additionally, multi-modal learning using foundational architectures like long short-term memory can also enable early predictions of exacerbations in COPD. By analyzing a range of modal data, including clinical data and radiographic information, these models capture potential disease features and trends. This helps doctors take timely intervention measures and reduce the risk of disease exacerbation. Deep learning has also shown promise in predicting hospital mortality rates. Through model training and optimization, the risk of patient mortality during hospital stays can be more accurately predicted. This is crucial for the rational allocation of hospital resources, prioritized patient care, and treatment decision-making^21,22^.

As previously described, research has demonstrated the potential achievements of artificial intelligence in the realm of healthcare services. Among them, conversational agents (CAs), also known as AI chatbots, are applications capable of communicating via natural language^23^. And with the rise of large language models, open AI’s generative pre-trained transformer (GPT) has gained prominence^24^. Trained on extensive text datasets, these models can generate natural language and be fine-tuned for a variety of language tasks^25^. For example, tasks such as language translation, text summarization, and text completion can be undertaken. After being trained on extensive conversational text datasets, these models can understand the context and intent of a conversation. They can be utilized as virtual assistants and other conversational interfaces that generate human-like responses in dialogue scenarios^26^. While initially mainly used for social chatting, these models are now increasingly being employed in specialized industries. Research in the field of intelligent diagnostics has demonstrated the feasibility of using large language models for radiological decision support in clinical settings, establishing them as effective tools for improving clinical workflows^27^. The isolation measures during the COVID-19 pandemic have heightened people’s awareness and understanding of online medical treatment. Doctors and patients have resorted to online consultations when face-to-face interactions are not possible. The number of remote consultations increased by 50%, further highlighting the importance of telemedicine^28^.

GPT, as a natural language processing model, serves various roles in healthcare. For instance, it can generate radiology reports to save radiologists time or assist in diagnostic decisions by providing differential and refined information. It can even communicate with patients, offering information regarding test results and follow-up treatment recommendations^29^. Additionally, some research has noted that digital therapy services provided by chatbots for mental health counseling have also gained recognition for their effectiveness^30^. Therefore, the future of healthcare will largely depend on the ability to accurately perform remote diagnoses. The collection of data remotely and its analysis through artificial intelligence will contribute to improved healthcare services and health outcomes. Regrettably, there is currently a relative paucity of research focusing on chronic disease auxiliary diagnosis based on large language models. Seizing this opportunity, our research aims to develop a system for aiding in the diagnosis of chronic diseases by leveraging large language models^31^. This aims to further enhance the quality of remote medical consultation services. This study is significant for the application of language models in healthcare and the development of systems that assist in the diagnosis of chronic diseases.

In the current technological context, this study comprehensively leverages the GPT-2 deep learning model. Through meticulous training, optimization, and packaging processes, we have successfully designed and developed an intelligent system for the auxiliary diagnosis of chronic diseases—Chat Ella. This system is capable of engaging in dialogues with patients, deeply inquiring into related symptoms, and thereby providing preliminary diagnostic results. Such an approach enables patients to gain an initial understanding of their health conditions and consult professional doctors in a timely manner when necessary. After a series of usability tests and evaluations on a validation set, including metrics such as accuracy and AUC, the system’s performance has met our anticipated standards. The chatbot-based medical auxiliary diagnosis model holds promise for broader promotion and application in the future.

The innovative aspects of this study are primarily reflected in the following areas:

- The study employs the GPT-2, a large-scale pre-trained language model, for in-depth learning on a rich corpus of disease symptom text, opening up new possibilities for remote diagnosis of chronic diseases.
- To allow users to more intuitively experience and utilize the training outcomes, we have specifically packaged the software and implemented a conversational interface, significantly enhancing the system’s interactivity and user-friendliness.
- Through computer usability questionnaire (CUQ) usability testing, we have not only ensured the functional implementation of the system but also further validated its practical utility and user satisfaction in real-world scenarios, guaranteeing the system’s efficiency and reliability.

## Methods

### Data collection

In this study, features were extracted from publicly available raw data acquired from Kaggle (https://www.kaggle.com/), an online platform for data science and machine learning competitions. Kaggle is recognized as a community dedicated to machine learning and data science, providing essential assets for a wide array of research activities^32–34^. Based on the data provided, a model was successfully constructed and trained, which was endowed with the capability to interpret patient symptom descriptions and assist in diagnosis. To ensure the model’s efficiency and accuracy, the data were meticulously organized and preprocessed, providing a solid foundation for the training and performance optimization of the Chat Ella model. In Supplementary Table 1, a detailed list of 24 common chronic disease categories relevant to diagnostic decision-making is presented. This dataset includes 1200 instances describing symptoms of chronic diseases. Additionally, a subset of symptom descriptions was randomly extracted and matched with corresponding disease outcomes; detailed cases are documented in Supplementary Table 2. Interested readers are directed to access the relevant raw datasets on kaggle.com.

### Construction, training, and evaluation of the auxiliary diagnosis model

Generative pre-trained transformer 2 (GPT-2) was released by open AI in 2019 as a model for natural language processing and generation. As a type of natural language generation model, GPT-2 employs a neural network architecture known as “transformer”. Represents an advancement of the transformer architecture, designed to generate coherent and realistic text through unsupervised learning from large datasets^35^. Owing to its efficiency in handling sequential data, it quickly became a mainstream technology in the field of natural language processing. The transformer model primarily relies on a self-attention mechanism, which enables the model to consider the relationships among different parts of the input sequence and generate output based on these relationships.

Developed based on several core principles, the pre-training and fine-tuning framework involves two stages: in the pre-training phase, the model is trained on a very large text dataset to learn a wide range of language patterns and structures. This stage is unsupervised, meaning it does not require manually labeled data. For training, GPT-2 utilizes a vast amount of text data collected from the internet, covering a wide range of topics and styles, which enables the model to understand and generate various forms of text. GPT-2 features an increased number of layers and parameters. For instance, includes 1.5 billion parameters and 48 transformer layers, enhancing its capability to comprehend complex texts and produce detailed responses. This allows the model to consider the relationships between every word in the input sequence during text generation, facilitating the production of more coherent and contextually relevant text. However, with the emergence of larger LLM models such as GPT-3, the advantages of this fine-tuning might diminish, especially in scenarios requiring higher generalizability^36^. Therefore, match this in our fine-tuning of GPT-2.

**Figure 1:**
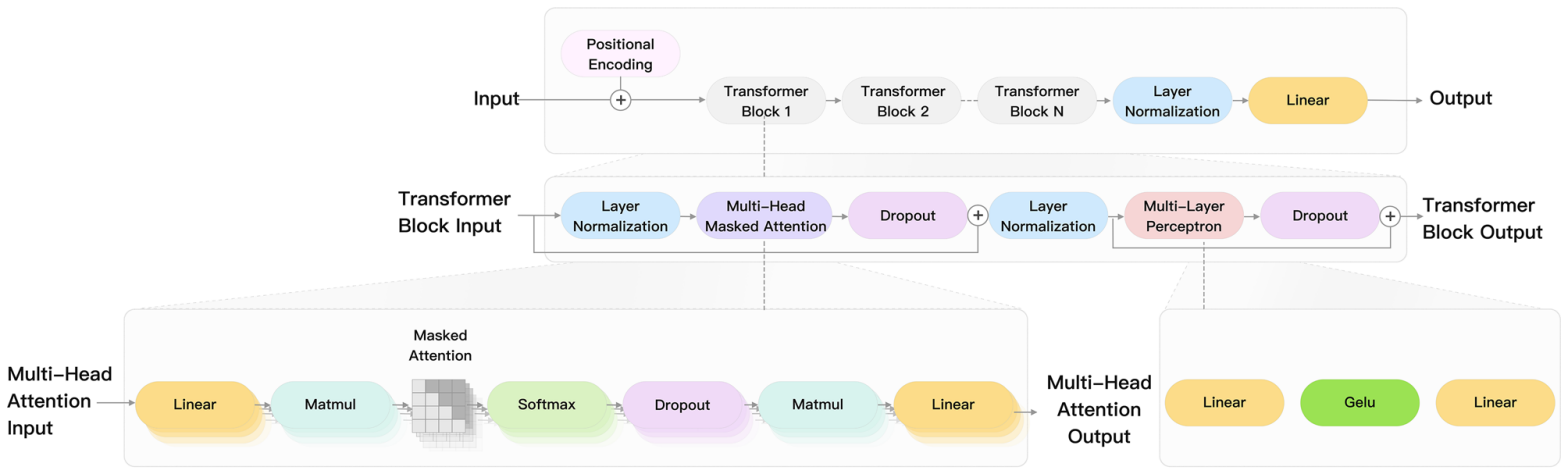
GPT-2 structure diagram.

The GPT-2 architecture is primarily composed of large transformer decoder blocks that integrate position encodings^37^. Within each decoder block, the multi-head self-attention mechanism and the multilayer perceptron are enveloped by layer normalization and dropout layers^38^. Time-series data inputted are organized into batches (samples, time steps, features) within the multi-head attention mechanism, wherein each time step is associated with a set of features. These features are partitioned and allocated to various processing units, enabling the simultaneous processing of different aspects of the input sequence and facilitating comprehensive analysis. This design of the architecture is shown to enhance the model’s capability to capture both short-term and long-term dependencies, thereby improving the prediction’s stability and accuracy. Illustrated in Fig. 1.

### Data preprocessing

During the data preprocessing stage, we initially carried out the following operations:

1. Deduplication: to ensure the quality and diversity of the data, all duplicate entries were removed from the dataset.
2. Text cleaning: irrelevant information, formatting errors, and special characters were removed from the text to ensure that each entry has a uniform and clear format.
3. Tokenization and encoding: to make the text data amenable to model processing, we tokenized the text and used specific encoding methods to convert it into numerical sequences that the model can recognize.

Through these steps, we obtained an optimized dataset, laying a solid foundation for the subsequent fine-tuning of the model.

### Model fine-tuning methods, training details, and parameter settings

In the domain of deep learning applications, fine-tuning has emerged as a prevalent strategy. This involves conducting additional training on a pre-trained model with a specialized dataset to enhance the model's performance for a specific task. For the purpose of this study, which aims to enable GPT-2 to diagnose patient symptoms more accurately, the model was fine-tuned. The objective was to predict potential chronic diseases based on descriptions of patient symptoms, constituting a multi-class classification task. The data employed for fine-tuning was sourced from a preprocessed medical dataset, where each entry comprises a patient’s symptom description paired with the corresponding disease label.

The selection of an appropriate loss function was deemed critical for successful training. Consequently, cross-entropy loss was chosen due to its suitability for multi-class classification tasks. To thoroughly evaluate the model's performance across various metrics, accuracy, recall, precision, F1 score, and AUC were set as the primary evaluation metrics. To promote the effectiveness and generalizability of the training, the dataset was partitioned into a training set and a test set. Parameter settings were acknowledged as vital during the training process. Owing to the robust performance of the Adam optimizer across diverse deep-learning tasks, it was selected for this training session. The initial learning rate was established at 3e-5, incorporating a strategy for learning rate decay throughout the training duration. The batch size was fixed at 32, which not only facilitated the effective utilization of GPU resources but also contributed to the stability of the training process. Moreover, a plan was devised to complete five full rounds of training. To mitigate the risk of overfitting, a dropout strategy was integrated into the model. In the training of GPT-2, convergence criteria were implemented to prevent overfitting and enhance computational efficiency. Specifically, an early stopping method was utilized, where the training process was terminated if the mean squared error (MSE) remained below a predetermined threshold for three consecutive epochs. This threshold was established at 0.5, determined based on the results from the optimization of the learning rate using the cyclical learning rate (CLR) scheduler^39–41^. This same threshold and count limit were maintained, with preliminary tests demonstrating that a threshold of 0.5 resulted in optimal model performance. Consequently, the dropout rate was also established at 0.5.

### Computational equipment and operating environment

All model training and evaluation for this study were conducted on a server equipped with an NVIDIA Tesla V100 GPU. This GPU, featuring 5120 CUDA cores and 16 GB of HBM2 memory, provided substantial computational support for large-scale deep learning models. The server ran on the Ubuntu 18.04 LTS operating system, noted for its stability and reliability. This operating system is widely used in the fields of big data and machine learning, benefiting from extensive open-source support and a robust community of resources. For the implementation and fine-tuning of the GPT-2 model, TensorFlow 2.4 was selected as the primary deep-learning framework. TensorFlow not only offers flexible capabilities for model design but also utilizes a powerful backend that maximizes the computational potential of the GPU. Additionally, Python libraries such as numpy and pandas were employed for data processing and analysis. For model serialization and storage, the h5py library was utilized.

### Integrated system design of Chat Ella

In the integrated system design of Chat Ella, the user input for symptom descriptions, database retrieval, and conversational responses must be tightly synchronized to provide timely and accurate feedback on medical symptoms to the users. The overall architecture of the system takes into account both backend processing and frontend user interface design, collaboratively ensuring exceptional user experience and system performance. The backend system primarily handles the processing of user input, database retrieval, and dialog responses. Its core task is to ensure seamless coordination among user query intents, database retrieval, and conversational responses, facilitating a smooth integration across these components. As for the frontend user interface, serving as the bridge for user interaction with Chat Ella, its design philosophy focuses on clarity, intuitiveness, and ease of use. Interface not only guides users in providing detailed symptom input but also clearly displays the responses and recommendations offered by Chat Ella. Furthermore, the tight integration between the front end and back end ensures that user inputs are accurately relayed to the back end, and feedback from the back end is presented to the user in a coherent manner.

### Backend system architecture

**Figure 2:**
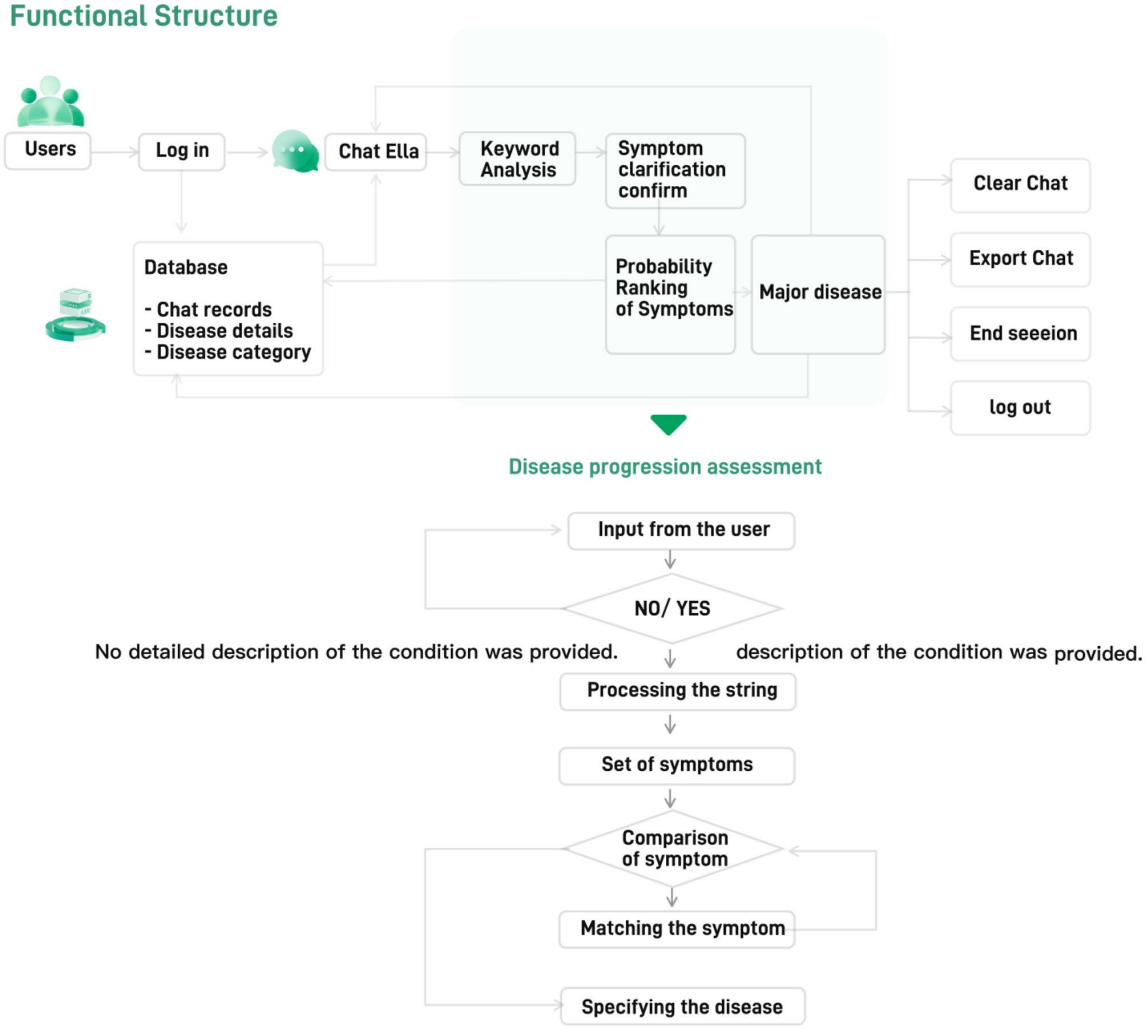
Overall system architecture of Chat Ella.

Chat Ella adopts a structured diagnostic approach based on the symptom descriptions provided by users. Initially, it optimally sorts the symptoms to ensure preliminary filtering based on their importance or prevalence. For each symptom description available in the database, Chat Ella is capable of real-time responses, facilitating real-time bi-directional communication with the user. Upon receiving a user query, the system retrieves relevant data from the database, processes it in the backend, and then sends it back to the user in the form of a message. The core of the diagnostic workflow lies in the comparative analysis between each symptom inputted by the user and the corresponding symptoms of common diseases stored in the database. Whenever a symptom aligns with a particular disease, that disease is added to a list of potential conditions. This process continues until all the symptoms described by the user have been matched. Upon the completion of the entire process, Chat Ella identifies the diseases that best match the user’s description, sorts them based on the probability of matching, and then provides the most likely disease information back to the user. This highly optimized workflow ensures the delivery of accurate and unambiguous diagnostic feedback to users within a short period, as illustrated in Fig. 2.

### Frontend user interface design and implementation

In consideration of visualizing backend data results and ensuring the integrity of the user experience, our objective was to minimize user friction while maintaining a universally interactive logic. The front end of Chat Ella was designed as a conversational interface, with the initial interface prototype created using Sketch version 83.1^42^, which allows for the creation of interactive interfaces. The final responsive interface was implemented in React^43^ as reported in recent advancements^44^. During development, the display sizes of user mobile devices were considered, allowing access from computers and tablets in addition to smartphones. For ease of user testing, the system was deployed on Tencent Cloud servers, eliminating the need for users to download any software to access the service.

### Usability testing

Between July 20 and July 28, 2023.Total of 64 adult participants responded to the survey online. Sample size meets the standards for similar usability surveys^45^.Collect feedback on the usability and acceptability of Chat Ella, such as adherence, user-friendliness, ease of use, appropriateness, user engagement, and user satisfaction, participants first interacted with Chat Ella and then answered a series of questions in the chatbot usability questionnaire (CUQ). Serves as a specific usability assessment tool for chatbots, CUQ is a usability survey questionnaire designed for chatbot assistants, similar in context to the system usability scale (SUS). However, given that our test subject is a conversational system, the use of SUS is not recommended. Better tailored for evaluating aspects related to chatbot personality, user experience, and error handling. Which is a widely used tool for evaluating system usability with a benchmark score of 68 out of 100. The CUQ consists of 16 questions and offers five response options, ranging from ‘strongly agree’ to ‘strongly disagree’. With scores calculated out of a total of 160, then normalized to 100. The CUQ evaluates aspects related to the chatbot’s personality, onboarding process, user experience, and error handling^46,47^. The supplementary material includes Table 3. Positive aspects of chatbot usability are assessed through odd-numbered questions, while negative aspects are assessed through even-numbered questions. Computational tools can be used to calculate each participant’s score and average scores^46^. As recommended, CUQ scores were further analyzed using SPSS 26.0 to produce statistical data. All participants provided written informed consent prior to participating in the study. All methods were carried out in accordance with relevant guidelines and regulations informed consent was obtained from all subjects.

### Ethical statement

Ethical review and approval were waived for this study due to the fact that the respondents were only required to answer questions without any control over humans. There is no relevant experimental research on the human body itself. (For this study due to the fact that the respondents were only required to answer questions without any control over humans). There is no relevant experimental research on the human body itself. It should also be emphasized that those participating in the chatbot usability testing are not patients. Participants were adults and voluntarily participated in the questionnaire written consent was informed. Because all 64 participants were of Chinese nationality, therefore, according to article 32 of the Measures for Ethical Review of Life Science and Medical Research Involving Human Beings, No. 4, “Ethical Review Measures for Life Science and Medical Research Involving Human Beings” of the People’s Republic of China, February 18, 2023, article 32: Ethical review may be exempted if it does not cause harm to human beings, or if it does not involve sensitive personal information or commercial interests. Therefore, review and approval by the Hanyang University Institutional Review Board is not required. This statement is intended to safeguard the ethical compliance of the research project and to protect the rights and privacy of the participants. The signature of the participant after reading the informed consent form will be displayed in the publication consent statement.

### Informed consent

Each participant has been made aware of the study and has only agreed to use the data in academic research.

## Result

### Results of the auxiliary diagnostic model

To validate the efficacy of the fine-tuned GPT-2 model in chronic disease diagnosis tasks, we relied on multiple evaluation metrics to assess the model’s performance. Below are the detailed assessment results.

As can be observed from Table 1, the model demonstrates outstanding overall performance on the test set. Particularly noteworthy is the area under the curve (AUC) value, which approaches 1, indicating a high discriminative capacity of the model in distinguishing different chronic diseases. Additionally, the accuracy, precision, recall, and F1 scores all exceeded 0.97, signifying that the model performs well both in predicting the correct disease categories and in distinguishing between positive and negative samples. These findings suggest that the fine-tuned GPT-2 model is not only capable of effectively processing and understanding patients’ symptom descriptions, but it can also offer highly reliable chronic disease diagnostic recommendations to medical personnel. This provides robust technical support for the future development of intelligent healthcare systems.

**Table 1:** Evaluation results of the auxiliary diagnostic mode.

Methods	Value
ACC	0.975
AUC	0.999
F1	0.974
Precision	0.977
Recall	0.975

### Backend development results

In the construction of the backend application for this research, the flask framework was employed, a choice known for its flexibility and simplicity in managing web applications. HTTP requests were effectively handled using the request module. Additionally, HTML templates were displayed and data was transmitted to the frontend by utilizing the render_template and jsonify modules. HTML rendering was facilitated by the render_template module, enabling dynamic web page generation based on backend data, while data formatting into JSON was accomplished using the jsonify module, enhancing the efficiency of data exchange between backend and frontend components.

### Model and tokenizer loading

**Figure 3:**
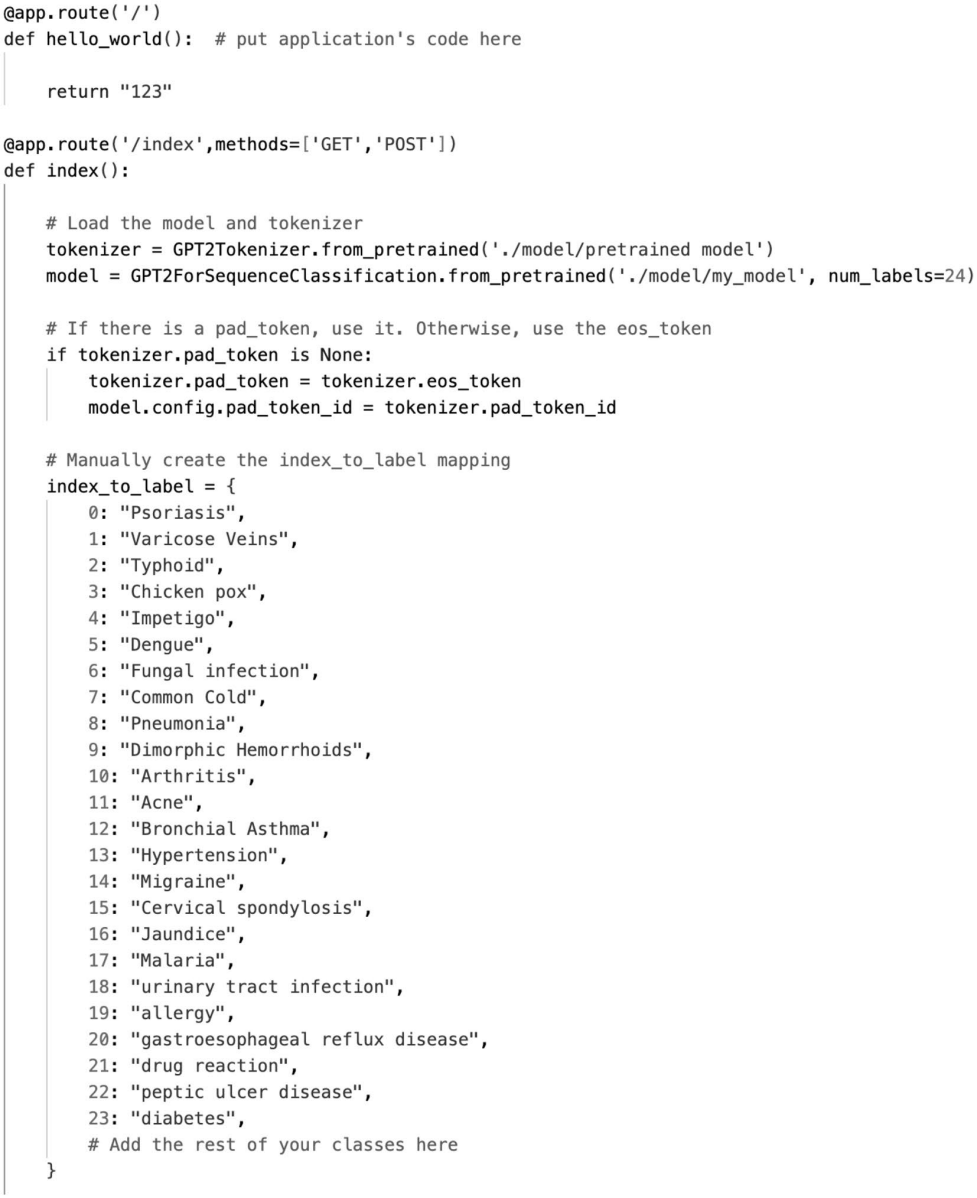
Disease condition tag classification.

In the main backend function, ‘index’, the pre-trained GPT-2 model along with its corresponding tokenizer (GPT-2 tokenizer and GPT-2 for sequence classification) were initially loaded from the transformer library. These models and tokenizers, specifically designed for text classification, assisted in establishing label mappings for disease tag classification, as depicted in Fig. 3.

### Disease label mapping and request handling

To precisely map the prediction results, a mapping from indices to disease labels, denoted as ‘index_to_label’, was constructed, where each index corresponds to a disease category. When processing frontend requests, the ‘index’ function retrieves parameters from the request, specifically the user-inputted symptom descriptions, which are utilized for model classification and disease category identification.

### Symptom description prediction

Upon obtaining the user-inputted symptom descriptions, the system employs the GPT-2 tokenizer to encode these descriptions, which are then used as inputs for classification prediction by the model. The output prediction result is a vector indicating the probabilities for each disease category.

### Result presentation and data feedback

**Figure 4:**
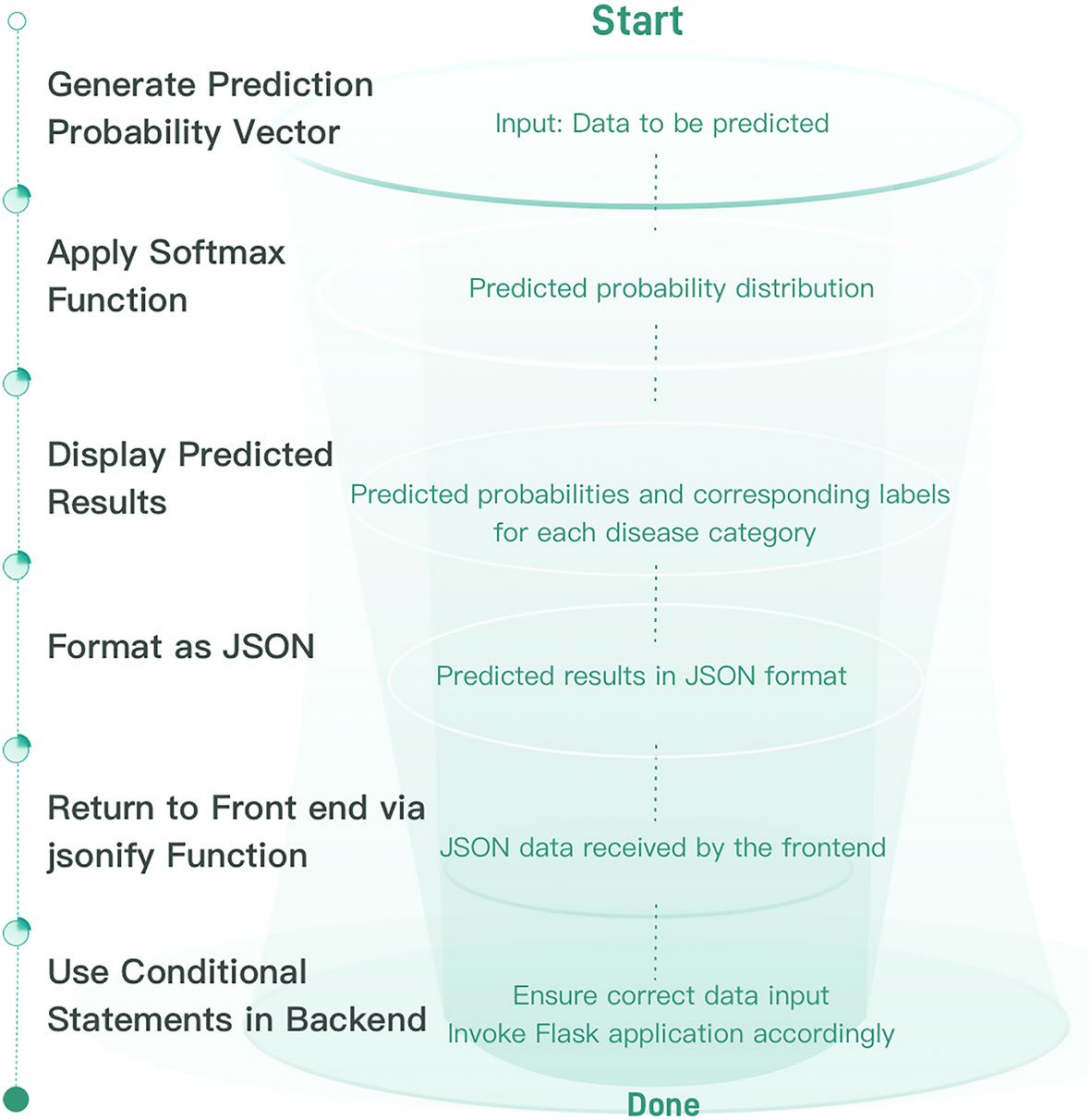
Disease prediction process.

The predicted probability vector is first processed through the softmax function to obtain the predicted probability distribution. Subsequently, the system displays the predicted probabilities for each disease category, along with their corresponding labels, to the user. The prediction results are formatted into JSON and returned to the frontend via the ‘jsonify’ function. In the final part of the backend, conditional statements are utilized to ensure correct data input, and the flask application is invoked accordingly. The entire process is depicted in Fig. 4.

### Frontend page design and implementation

**Figure 5:**
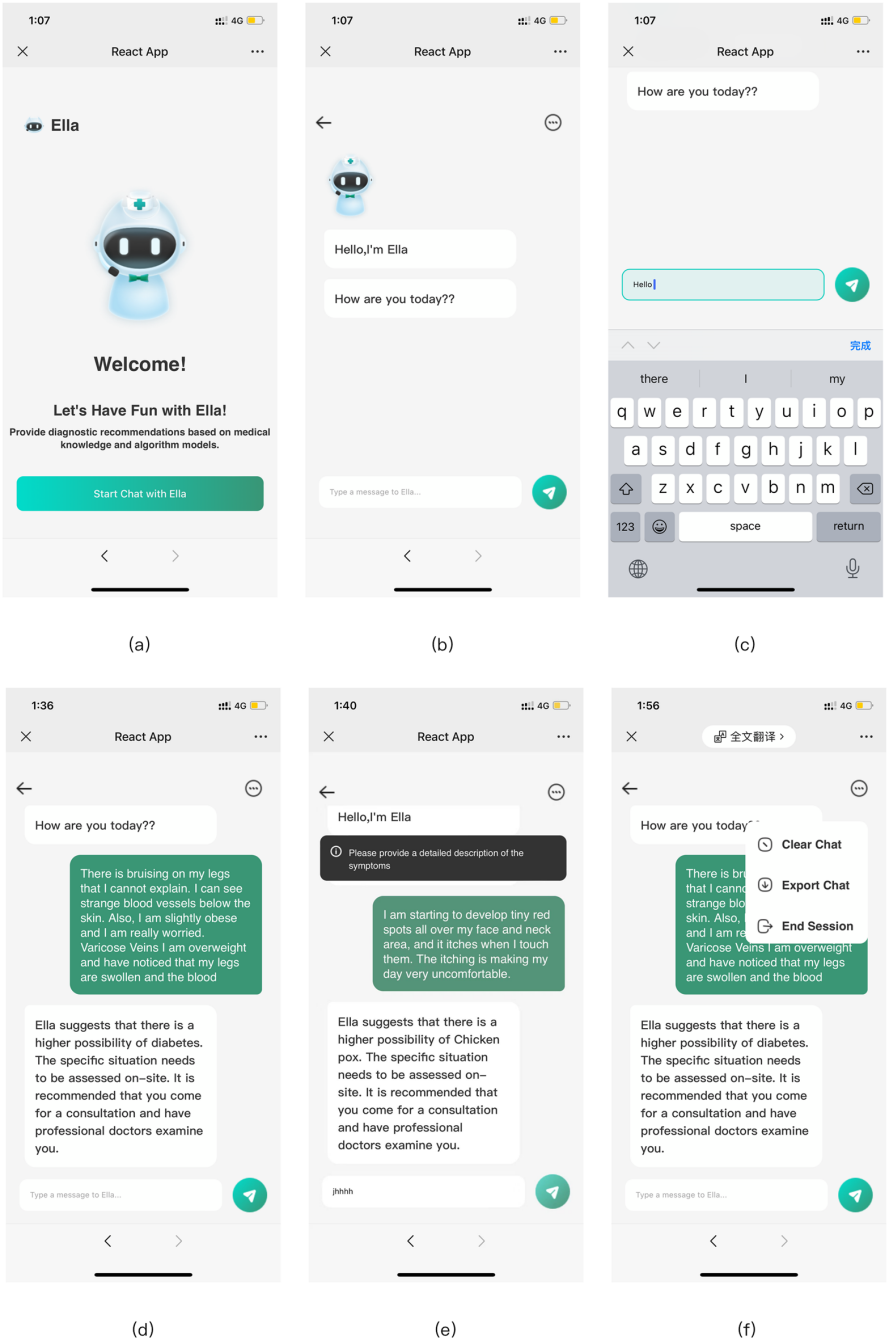
Chat Ella dialogue system user interface design screenshot.

React components were used to design and implement the user interface. Emotion and user sentiment have been consistently regarded as lasting and effective key factors in user experience design^48,49^. In light of this, a virtual persona for the Ella chatbot was built and displayed on the page to more intuitively guide user interaction, as illustrated in Fig. 4a, b. Users can directly input their symptom descriptions into the interface and interact with the Chat Ella chatbot to receive relevant responses and suggestions. React’s functional components, along with a series of Hooks, were utilized to facilitate this interaction^50^, such as ‘useState’, ‘useRef’, and ‘useEffect’, were employed to manage component state and references. Several state variables, such as ‘showMenu’, ‘inputTxt’, and ‘messages’, were established within the components. These variables are used to control the chat interface display and manage user input. The ‘useRef’ hook was also used to create references to DOM elements, such as ‘chatDiv’ and ‘menuRef’, which are essential for subsequent operations. Additionally, several event-handling functions, such as ‘onMenuClick’, ‘onBackClick’, and ‘onSendClick’, were integrated into the component to manage various user actions. To ensure chat interface could display the latest messages in real-time, ‘useEffect’ was employed to monitor changes to the messages array, and the scroll position of the chat interface was accordingly set, as depicted in Fig. 5a–c. The ‘axios’ library was utilized to implement HTTP communication with the backend server. When a user enters symptom descriptions in the chat interface and clicks the send button, the system evaluates their input and adds it to the messages array, provide recommendations for the optimal correspondence, as shown in Fig. 5d. If no symptom-related statements are detected, a prompt is displayed, suggesting that the user provide more detailed symptom descriptions, as shown in Fig. 5e. Furthermore, the chat interface features a menu option. When users click on the menu icon, they can view or hide various menu options, such as deleting records and exporting logs, as depicted in Fig. 5f. The server’s link is http://62.234.219.85:8080/, and the detailed source code has been packaged and stored in the supplementary materials.

### Quantitative analysis of usability results

A total of 64 adult participants, comprising 36 females and 28 males, responded to the survey. The educational background of the participants included 41 with a bachelor’s degree, eight with a master’s degree, and three with a Ph.D., as shown in Table 2.

**Table 2:** Characteristics of the survey respondents (n = 64).

Education group	Female	Gender male	Total
Bachelor	25 (60.98%)	16 (39.02%)	41 (64.06%)
Master	8 (57.14%)	6 (42.86%)	14 (21.87%)
Doctoral	3 (33.33%)	6 (66.67%)	9 (14.06%)
Total	36 (56.25%)	28 (43.75%)	64 (100%)

#### CUQ findings

**Figure 6:**
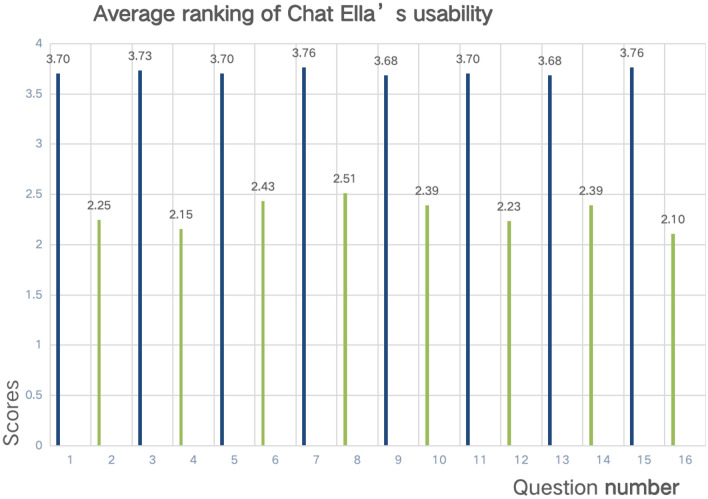
Average ranking of chatbot usability questionnaire.

The odd-numbered questions focus on the positive aspects of the chatbot experience. Among the positive feedback regarding Chat Ella’s usability, the question “The chatbot is user-friendly during the initial setup” (Q7) and “It is easy to use” (Q15) scored the highest with an average rating of 3.76. The lowest average scores for positive questions were for questions 1, 5, and 11, with an average score of 3.70. The even-numbered questions focus on the negative aspects. For instance, question 8 “Using the chatbot can sometimes be confusing” scored the highest among these with an average rating of 3.4. On the other hand, question 16, which asked if “Using the chatbot is complicated,” received the lowest average score of 2.1, as shown in Fig. 6. Among the 64 participants, male respondents had a higher average CUQ score of 68.69 compared to females. Participants with a Ph.D. had the highest average score, registering at 82.24. The average score for those who interacted via tablets was higher (72.48) than for those who used other devices. Those who believed that Chat Ella could diagnose diseases had an average score of 66.41. The overall average score across all participants was 68.31, as shown in Table 3. These results indicate a generally positive perception of Chat Ella’s usability, though there is room for improvement, particularly in aspects that received lower scores. These insights are valuable for future updates and refinements to the system.

**Table 3:** Chatbot usability questionnaire scores for study participants (n = 64) according to participant characteristics.

	Mean	SD	Minimum	Maximum	SE
Gender					
Female	66.57	28.43	14.06	95	4.73
Male	68.69	26.59	14.06	93.75	5.02
Education					
Bachelor	66.69	27.71	14.06	87.5	4.32
Master	60.38	32.57	14.06	93.75	8.7
Doctorate	82.25	5.59	75	95	1.86
Interactive device					
Computer	60.24	31.88	14.06	87.5	7.51
Tablet computer	72.48	24.89	18.75	93.75	5.86
Phone (Ios)	68.3	26.42	18.75	95	6.22
Phone (android)	70.15	26.45	17.19	87.5	8.36
Chat Ella can provide auxiliary diagnosis					
Yes	66.41	28.77	14.06	87.5	6.43
No	66.31	27.91	14.06	95	5.18
Maybe	71.25	26.25	18.75	93.75	6.77

## Discussion

Amidst rapid advancements in information technology, artificial intelligence (AI) has undergone extensive validation and is increasingly gaining prominence. This trend is particularly notable in the healthcare sector, where AI-assisted medical services are receiving widespread attention and adoption, especially for specific use cases that benefit from efficient information retrieval. In this context, a chatbot named Chat Ella, grounded in large language models and deep learning techniques, was developed. Integrated with a comprehensive medical database, this chatbot is capable of accurately interpreting patients’ symptom descriptions and medical histories, subsequently providing diagnostic recommendations. The GPT-2 pre-trained model was employed for system development. To evaluate the practical application of the system, a usability assessment was conducted for users of Chat Ella. The results indicate that the average CUQ score among the participants reached 68.31, significantly surpassing general usability evaluation benchmarks. This indicates that Chat Ella has significant potential to assist in the auxiliary diagnosis of chronic diseases remotely and in a non-face-to-face manner.

The primary advantage of this system lies in its ability to conduct precise disease detection and matching based on user-provided symptom descriptions. This approach not only offers convenience but also ensures that patients do not overlook symptoms that might seem less significant, thereby providing them with a simple, efficient, and accurate means to understand their health status without the need for hospital visits or blind internet searches for symptoms. It is anticipated that this approach can not only effectively alleviate the issue of unequal distribution of medical resources but also enhance the work efficiency of medical professionals, thus offering broad application prospects in the healthcare sector.

In the selection of an appropriate language model to assist in diagnostic research, although advanced models such as GPT-3 and updated versions of Chat GPT are available in the current landscape of deep learning, GPT-2 was chosen for study. This decision was based on multiple considerations. Firstly, GPT-2 is recognized for its computational and storage efficiency while offering a level of accuracy comparable to larger models, thus ensuring resource efficiency. Secondly, its smaller scale is associated with better model interpretability and stability, which are critical in healthcare applications. Additionally, the prior release and widespread utilization of GPT-2 mean that its performance on a variety of tasks has been extensively validated, providing a reliable foundation for our research. GPT-2 is also readily fine-tunable, which allows for flexible adaptation to specific diagnostic tasks. Lastly, the adoption of GPT-2 simplifies the deployment process. Therefore, GPT-2 was found to offer an optimal balance for this study.

This study does indeed have certain limitations. First, the dataset we collected is limited in size, necessitating further expansion to ensure the model’s generalization capabilities. Second, the current chatbot primarily supports English interaction, and its response patterns are relatively fixed. In our usability testing, the majority of the participants in the study hail from academic and professional backgrounds in digital design and computer science, as well as clinical medicine. These participants engaged in tests focusing on their personally diagnosed medical experiences in the past which may limit the applicability of our findings to a broader user base. It’s worth noting that although our research focuses on the usability of a prototype model for assisting in the diagnosis of chronic diseases, aiming to save development time while ensuring product quality, some issues might still arise in practical applications. GPT-2 being a large language model that was pre-trained on extensive datasets, as a black box system, its reasoning processes cannot be fully explained or audited. Therefore, it is crucial to conduct extensive validation and close monitoring before its clinical adoption^51^. Additionally, the knowledge contained within it is only reflective up to the point when the training data was collected, which means it may not provide accurate information or insights about developments that occurred after the data collection period. User may use non-standard or informal language to describe their symptoms, potentially making it difficult for the chatbot to accurately interpret and respond.

To address these challenges and limitations. In deed to address these limitations, it is essential to refine and optimize training datasets to reduce biases and inaccuracies. Integrating external knowledge bases or fact-checking modules within the model can verify the accuracy of generated content. Human–machine interaction methods supervised by clinical physicians and featuring feedback mechanisms can continuously improve clinical logic. Further enrich and refine our database and optimize related algorithms to improve diagnostic accuracy and adaptability in responding to questions. Accessing updated databases or employing real-time fact-checking tools can aid the model in making more accurate judgments and generating precise information. Enhancing the model’s contextual understanding ability is crucial for better grasping complex situations and incorporating relevant new information.

## Conclusion

This study has constructed an innovative question–answering system for chronic medical conditions, based on large language models. As large language models continue to advance, the focus on how to effectively integrate them into clinical workflows to enhance rather than replace human decision-making becomes particularly important. The system incorporates an extensive corpus of medical knowledge and is designed to develop a chatbot for intelligent diagnosis of common chronic diseases. Empirical results from a CUQ test demonstrated the system’s anticipated benefits and received positive feedback on usability. While current AI technology cannot fully replace the functions of a medical professional. The widespread application of chatbot technology in healthcare not only enhances the patient experience, expedites medical service response time, and boosts overall service efficiency, but also lowers healthcare economic costs. This technology offers an efficient and convenient option for patients who prefer or are unable to engage in face-to-face consultations.

Looking forward, considering upgrading the system to support voice input and image recognition features. High-quality training data is pivotal for the support of voice and image recognition technologies. This study sought support from medical collaborations to collect diverse training data covering chronic disease types and varied conditions in language and imagery. Data augmentation techniques were employed to enhance the model’s generalization capabilities. The model was integrated into an existing question-and-answer system, employing a modular system architecture designed to facilitate data exchange and function integration across different systems using application programming interfaces multimodal data fusion was crucial, effectively integrating information from various data sources including text, voice, and images, which is essential for improving the system’s diagnostic accuracy and response relevance. Fusion network strategies in deep learning ensured the effective integration of data across different modalities. Such enhancements will not only further improve the system’s usability but will also allow it to handle a wider variety of data types, thereby facilitating further doctor appointments following a chronic disease diagnosis. Leveraging deep learning and large language models, the application prospects of this technology extend beyond merely aiding in the diagnosis of chronic diseases. These technologies have the potential to be widely deployed in the design and development of various medical systems.

### Supplementary Information

Supplementary Tables.

Supplementary Information.

## Data Availability

The raw data used during the current study are available from the corresponding author upon reasonable request.
