# Supplementary material for: A chatbot based question and answer system for the auxiliary diagnosis of chronic diseases based on large language model
Source: Sci Rep. 2024 Jul 25;14:17118. doi: 10.1038/s41598-024-67429-4 (PMC11272932; doi:10.1038/s41598-024-67429-4)
Supplement: Supplementary file 1 — Supplementary Tables. [file 41598_2024_67429_MOESM1_ESM.docx]

**Table 1 List of diagnostic decisions**

| **Disease category** |
| --- |
| Psoriasis |
| Varicose Veins |
| Typhoid |
| Chicken pox |
| Impetigo |
| Dengue |
| Fungal infection |
| Common Cold |
| Pneumonia |
| Dimorphic Hemorrhoids |
| Arthritis |
| Acne |
| Bronchial Asthma |
| Hypertension |
| Migraine |
| Cervical spondylosis |
| Jaundice |
| Malaria |
| urinary tract infection |
| allergy |
| gastroesophageal reflux disease |
| drug reaction |
| peptic ulcer disease |
| diabetes |

**Table 2 Training data set of symptom description cases.**

| **Symptom Description** | **Disease category** |
| --- | --- |
| I have been experiencing a skin rash on my arms, legs, and torso for the past few weeks. It is red, itchy,  and covered in dry, scaly patches. | Psoriasis |
| There is bruising on my legs that I cannot explain. I can see strange blood vessels below the skin. Also, I am slightly obese and I am really worried. | Varicose Veins |
| I've lost my appetite and am always sick. I'm also developing a mild fever. Also, my abdominal part pains a lot. I don't know what the reason behind all of these is. | Typhoid |
| I am starting to develop tiny red spots all over my face and neck area, and it itches when I touch them. The itching is making my day very uncomfortable. | Chicken pox |
| I'm having problems breathing and have a very high fever. I'm perspiring a lot and experiencing extreme fatigue and chilly. My heart is racing, and I'm coughing up some brownish mucus. | Pneumonia |
| Frequent urges to urinate with little output, pain during urination, cloudy or bloody urine, strong or foul-smelling urine, pelvic pain, low fever, nausea and vomiting | Urinary tract infection |

**Table3 Chat Ella Usability Testing Survey**

| *Please confirm: this questionnaire is voluntary. After filling out, it is deemed that the information in it can be used for research on Chat Ella usuability test. | | | | | |
| --- | --- | --- | --- | --- | --- |
| 1. What is your gender? | | | |  | |
| Female | | | |  |  |
| Male | | | |  |  |
| 2. What is your Education? | | | |  |  |
| Bachelor | | | |  |  |
| Master | | | |  |  |
| Doctoral | | | |  |  |
| 3. How did you interact with Chat Ella? | | | |  |  |
| Computer | | | |  |  |
| Tablet computer | | | |  |  |
| Phone（Ios） | | | |  |  |
| Phone（Android ） | | | |  |  |
| 4. Do you think Chat Ella could help you assisted diagnosis ？ | | | |  |  |
| Yes | | | |  |  |
| No | | | |  |  |
| Maybe | | | |  |  |
| 5. Chatbot Usability Questionnaire (Holmes et al. 2019) |  | | | | |
| Q1 The chatbot’s personality was realistic and engaging | 5 | 4 | 3 | 2 | 1 |
| Q2 The chatbot seemed too robotic | 5 | 4 | 3 | 2 | 1 |
| Q3 The chatbot was welcoming during initial setu | 5 | 4 | 3 | 2 | 1 |
| Q4 The chatbot seemed very unfriendly | 5 | 4 | 3 | 2 | 1 |
| Q5 The chatbot explained its scope and purpose well | 5 | 4 | 3 | 2 | 1 |
| Q6 The chatbot gave no indication as to its purpose | 5 | 4 | 3 | 2 | 1 |
| Q7 The chatbot was easy to navigate | 5 | 4 | 3 | 2 | 1 |
| Q8 It would be easy to get confused when using the chatbot | 5 | 4 | 3 | 2 | 1 |
| Q9 The chatbot understood me well | 5 | 4 | 3 | 2 | 1 |
| Q10 The chatbot failed to recognise a lot of my input | 5 | 4 | 3 | 2 | 1 |
| Q11 Chatbot responses were useful, appropriate, and informative | 5 | 4 | 3 | 2 | 1 |
| Q12 Chatbot responses were not relevant | 5 | 4 | 3 | 2 | 1 |
| Q13 The chatbot coped well with any errors or mistakes | 5 | 4 | 3 | 2 | 1 |
| Q14 The chatbot seemed unable to handle any errors | 5 | 4 | 3 | 2 | 1 |
| Q15 The chatbot was very easy to use | 5 | 4 | 3 | 2 | 1 |
| Q16 The chatbot was very complex | 5 | 4 | 3 | 2 | 1 |
