# Supplementary material for: A chatbot based question and answer system for the auxiliary diagnosis of chronic diseases based on large language model
Source: Sci Rep. 2024 Jul 25;14:17118. doi: 10.1038/s41598-024-67429-4 (PMC11272932; doi:10.1038/s41598-024-67429-4)
Supplement: Supplementary file 2 — Supplementary Information. [file 41598_2024_67429_MOESM2_ESM.zip › coding file/clienth5/build/index.html]

React App You need to enable JavaScript to run this app.
