# Supplementary figures and images for: A chatbot based question and answer system for the auxiliary diagnosis of chronic diseases based on large language model
Source: Sci Rep. 2024 Jul 25;14:17118. doi: 10.1038/s41598-024-67429-4 (PMC11272932; doi:10.1038/s41598-024-67429-4)

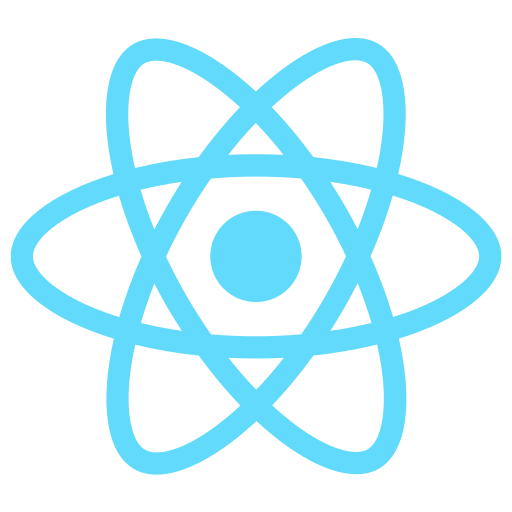

Supplement: Supplementary file 2 — Supplementary Information. [file 41598_2024_67429_MOESM2_ESM.zip › coding file/clienth5/public/logo512.png]

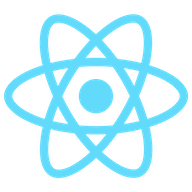

Supplement: Supplementary file 2 — Supplementary Information. [file 41598_2024_67429_MOESM2_ESM.zip › coding file/clienth5/public/logo192.png]

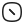

Supplement: Supplementary file 2 — Supplementary Information. [file 41598_2024_67429_MOESM2_ESM.zip › coding file/clienth5/src/img/clearChat1.png]

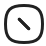

Supplement: Supplementary file 2 — Supplementary Information. [file 41598_2024_67429_MOESM2_ESM.zip › coding file/clienth5/src/img/clearChat2.png]

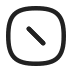

Supplement: Supplementary file 2 — Supplementary Information. [file 41598_2024_67429_MOESM2_ESM.zip › coding file/clienth5/src/img/clearChat3.png]

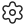

Supplement: Supplementary file 2 — Supplementary Information. [file 41598_2024_67429_MOESM2_ESM.zip › coding file/clienth5/src/img/setup1.png]

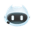

Supplement: Supplementary file 2 — Supplementary Information. [file 41598_2024_67429_MOESM2_ESM.zip › coding file/clienth5/src/img/headSay1.png]

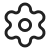

Supplement: Supplementary file 2 — Supplementary Information. [file 41598_2024_67429_MOESM2_ESM.zip › coding file/clienth5/src/img/setup2.png]

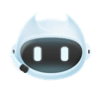

Supplement: Supplementary file 2 — Supplementary Information. [file 41598_2024_67429_MOESM2_ESM.zip › coding file/clienth5/src/img/headSay3.png]

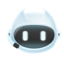

Supplement: Supplementary file 2 — Supplementary Information. [file 41598_2024_67429_MOESM2_ESM.zip › coding file/clienth5/src/img/headSay2.png]

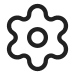

Supplement: Supplementary file 2 — Supplementary Information. [file 41598_2024_67429_MOESM2_ESM.zip › coding file/clienth5/src/img/setup3.png]

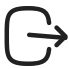

Supplement: Supplementary file 2 — Supplementary Information. [file 41598_2024_67429_MOESM2_ESM.zip › coding file/clienth5/src/img/endSession3.png]

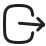

Supplement: Supplementary file 2 — Supplementary Information. [file 41598_2024_67429_MOESM2_ESM.zip › coding file/clienth5/src/img/endSession2.png]

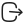

Supplement: Supplementary file 2 — Supplementary Information. [file 41598_2024_67429_MOESM2_ESM.zip › coding file/clienth5/src/img/endSession1.png]

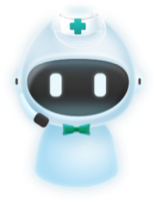

Supplement: Supplementary file 2 — Supplementary Information. [file 41598_2024_67429_MOESM2_ESM.zip › coding file/clienth5/src/img/headBig1.png]

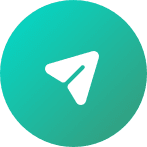

Supplement: Supplementary file 2 — Supplementary Information. [file 41598_2024_67429_MOESM2_ESM.zip › coding file/clienth5/src/img/send3.png]

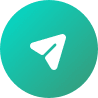

Supplement: Supplementary file 2 — Supplementary Information. [file 41598_2024_67429_MOESM2_ESM.zip › coding file/clienth5/src/img/send2.png]

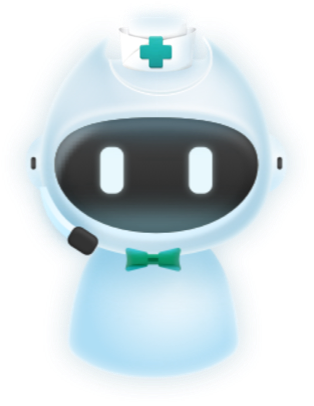

Supplement: Supplementary file 2 — Supplementary Information. [file 41598_2024_67429_MOESM2_ESM.zip › coding file/clienth5/src/img/headBig2.png]

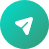

Supplement: Supplementary file 2 — Supplementary Information. [file 41598_2024_67429_MOESM2_ESM.zip › coding file/clienth5/src/img/send1.png]

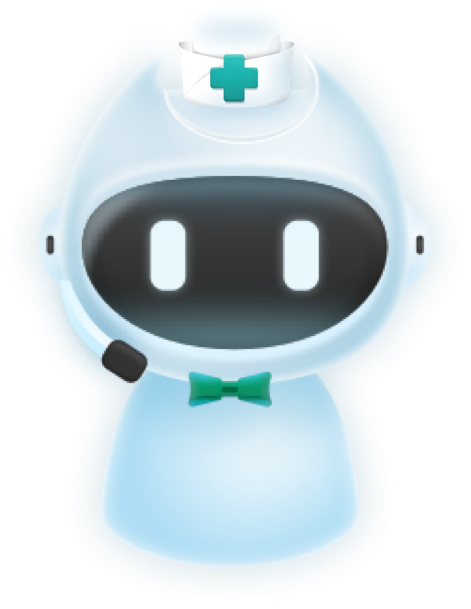

Supplement: Supplementary file 2 — Supplementary Information. [file 41598_2024_67429_MOESM2_ESM.zip › coding file/clienth5/src/img/headBig3.png]

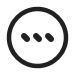

Supplement: Supplementary file 2 — Supplementary Information. [file 41598_2024_67429_MOESM2_ESM.zip › coding file/clienth5/src/img/menu3.png]

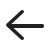

Supplement: Supplementary file 2 — Supplementary Information. [file 41598_2024_67429_MOESM2_ESM.zip › coding file/clienth5/src/img/back2.png]

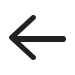

Supplement: Supplementary file 2 — Supplementary Information. [file 41598_2024_67429_MOESM2_ESM.zip › coding file/clienth5/src/img/back3.png]

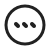

Supplement: Supplementary file 2 — Supplementary Information. [file 41598_2024_67429_MOESM2_ESM.zip › coding file/clienth5/src/img/menu2.png]

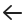

Supplement: Supplementary file 2 — Supplementary Information. [file 41598_2024_67429_MOESM2_ESM.zip › coding file/clienth5/src/img/back1.png]

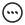

Supplement: Supplementary file 2 — Supplementary Information. [file 41598_2024_67429_MOESM2_ESM.zip › coding file/clienth5/src/img/menu1.png]

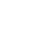

Supplement: Supplementary file 2 — Supplementary Information. [file 41598_2024_67429_MOESM2_ESM.zip › coding file/clienth5/src/img/warning2.png]

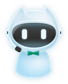

Supplement: Supplementary file 2 — Supplementary Information. [file 41598_2024_67429_MOESM2_ESM.zip › coding file/clienth5/src/img/headSmall1.png]

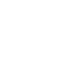

Supplement: Supplementary file 2 — Supplementary Information. [file 41598_2024_67429_MOESM2_ESM.zip › coding file/clienth5/src/img/warning3.png]

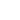

Supplement: Supplementary file 2 — Supplementary Information. [file 41598_2024_67429_MOESM2_ESM.zip › coding file/clienth5/src/img/warning1.png]

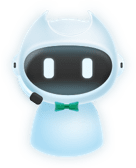

Supplement: Supplementary file 2 — Supplementary Information. [file 41598_2024_67429_MOESM2_ESM.zip › coding file/clienth5/src/img/headSmall2.png]

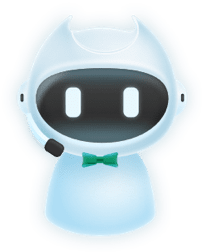

Supplement: Supplementary file 2 — Supplementary Information. [file 41598_2024_67429_MOESM2_ESM.zip › coding file/clienth5/src/img/headSmall3.png]

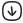

Supplement: Supplementary file 2 — Supplementary Information. [file 41598_2024_67429_MOESM2_ESM.zip › coding file/clienth5/src/img/exportChat1.png]

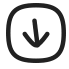

Supplement: Supplementary file 2 — Supplementary Information. [file 41598_2024_67429_MOESM2_ESM.zip › coding file/clienth5/src/img/exportChat3.png]

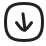

Supplement: Supplementary file 2 — Supplementary Information. [file 41598_2024_67429_MOESM2_ESM.zip › coding file/clienth5/src/img/exportChat2.png]
